# Effect of Obesity on the Use of Antiarrhythmics in Adults With Atrial Fibrillation: A Narrative Review

**DOI:** 10.1002/clc.24336

**Published:** 2024-08-21

**Authors:** Fahad Shaikh, Rochelle Wynne, Ronald L. Castelino, Patricia M. Davidson, Sally C. Inglis, Caleb Ferguson

**Affiliations:** ^1^ Centre for Chronic & Complex Care Research Blacktown Hospital, Western Sydney Local Health District Blacktown New South Wales Australia; ^2^ School of Nursing, Faculty of Science, Medicine & Health University of Wollongong Wollongong New South Wales Australia; ^3^ School of Nursing & Midwifery, Centre for Quality & Patient Safety in the Institute for Health Transformation Deakin University Burwood Victoria Australia; ^4^ Deakin‐Western Health Partnership Western Health St Albans Victoria Australia; ^5^ Faculty of Medicine and Health University of Sydney Camperdown New South Wales Australia; ^6^ Pharmacy Department Blacktown Hospital, Western Sydney Local Health District Blacktown New South Wales Australia; ^7^ University of Wollongong Wollongong New South Wales Australia; ^8^ School of Nursing Johns Hopkins University Baltimore Maryland USA; ^9^ Improving Palliative, Aged and Chronic Care through Clinical Research and Translation (IMPACCT) University of Technology Sydney Sydney New South Wales Australia

**Keywords:** antiarrhythmic, atrial fibrillation, body mass index, drug dosage, obesity, weight

## Abstract

**Background:**

Atrial fibrillation (AF) and obesity coexist in approximately 37.6 million and 650 million people globally, respectively. The anatomical and physiological changes in individuals with obesity may influence the pharmacokinetic properties of drugs.

**Aim:**

This review aimed to describe the evidence of the effect of obesity on the pharmacokinetics of antiarrhythmics in people with AF.

**Methods:**

Three databases were searched from inception to June 2023. Original studies that addressed the use of antiarrhythmics in adults with AF and concomitant obesity were included.

**Results:**

A total of 4549 de‐duplicated articles were screened, and 114 articles underwent full‐text review. Ten studies were included in this narrative synthesis: seven cohort studies, two pharmacokinetic studies, and a single case report. Samples ranged from 1 to 371 participants, predominately males (41%–85%), aged 59–75 years, with a body mass index (BMI) of 23–66 kg/m^2^. The two most frequently investigated antiarrhythmics were amiodarone and dofetilide. Other drugs investigated included diltiazem, flecainide, disopyramide, propafenone, dronedarone, sotalol, vernakalant, and ibutilide. Findings indicate that obesity may affect the pharmacokinetics of amiodarone and sodium channel blockers (e.g., flecainide, disopyramide, and propafenone). Factors such as drug lipophilicity may also influence the pharmacokinetics of the drug and the need for dose modification.

**Discussion:**

Antiarrhythmics are not uniformly affected by obesity. This observation is based on heterogeneous studies of participants with an average BMI and poorly controlled confounding factors such as multimorbidity, concomitant medications, varying routes of administration, and assessment of obesity. Controlled trials with stratification at the time of recruitment for obesity are necessary to determine the significance of these findings.

## Graphical abstract

This study described the effect of obesity on the pharmacokinetics of antiarrhythmics in AF patients. After screening 4549 articles, 10 studies were included. Results indicated that amiodarone and sodium channel blockers are most affected by obesity. Further controlled trials are needed to validate these findings.

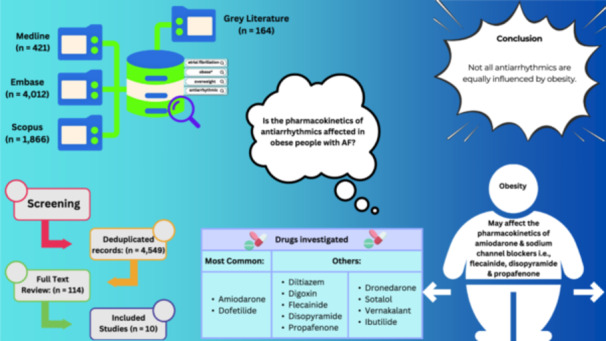

## 1 Background

Atrial fibrillation (AF) is the most common type of sustained cardiac arrhythmia, affecting approximately 37.6 million people [1]. The incidence and prevalence of AF have been increasing over time and are projected to double by 2030 across several regions, such as Europe, the United States, and Australia [2, 3, 4, 5, 6]. AF has been previously reported to be higher in high‐income countries with a high sociodemographic index [7, 8]. It is hypothesized that increasing incidence is secondary to an ageing global population and the increasing prevalence of diabetes, cardiovascular disease, and risk factors such as hypertension, atherosclerosis, and obesity [2, 7].

Obesity is a complex, multifactorial, and largely preventable condition that has steadily increased to epidemic proportions [9, 10]. According to the World Health Organization (WHO), the global prevalence of obesity has surged to 890 million adults in 2022 [11] and is projected to further increase to 1.12 billion by 2030 [12].

Although the body mass index (BMI) remains the prevalent means of gauging obesity levels, including by the WHO, it often overlooks crucial factors such as waist circumference, fat percentage, build, lifestyle, and body fat distribution/body composition, thus leading to an ambiguity in its use as a biomarker of “unhealthiness” [13]. Nevertheless, in most cases, it can help ascertain if an individual is truly obese or overweight [14].

The prevalence of obesity is higher among high‐income countries and is relatively similar across both sexes [15]. In developed or high‐income countries, obesity disproportionally affects individuals from lower socioeconomic groups, particularly in the female population [16]. The reason for this difference is not completely understood, but it is commonly believed that the increased availability and accessibility of low‐cost, highly processed, calorie‐dense foods in lower socioeconomic areas, compared to more affluent areas, underpins this problem [17]. However, there is limited evidence regarding this association, suggesting that other unknown influential factors may exist [16].

Despite obesity being an emergent risk factor for AF, the underlying mechanisms of its relationship with AF are not well understood, yet are known to be multifactorial including hemodynamical, inflammatory, and metabolic changes [18, 19, 20].

The pharmacokinetics of a drug are arguably the most important aspect of its lifecycle and relevant measures investigated are absorption, distribution, metabolism, and clearance/elimination (ADME). In obese adults, the anatomical and physiological changes can affect distribution (volume of distribution, Vd) and clearance, which in turn impacts elimination half‐life [21, 22]. In addition to ADME measures, another important factor that influences ADME is the drug's level of lipophilicity [23]. Bruno et al. showed that lipophilic drugs disproportionately distribute into the adipose tissue in obese adults, thus leading to inconsistent prolongation of elimination half‐life [23]. Recent research [24] has shown that drugs with high Vd and lipophilic properties display significantly different pharmacokinetics in obese individuals compared to those of normal weight. Although it is hypothesized that these parameters are mainly affected in patients who are morbidly or extremely obese (class III), these considerations should not be overlooked or ignored [21, 22].

Globally, clinical practice guidelines for AF recommend prophylactic anticoagulants and rate and/or rhythm control medications [25]. Landmark trials such as the AFFIRM [26] and HOT CAFE [27] trials have shown that survival associated with rate control is comparable to cardioversion and antiarrhythmic drugs (HR, 1.15 [95% CI, 0.99–1.34]; *p* = 0.08) and a composite of all‐cause mortality, thromboembolic events, or major bleeding (OR, 1.98 [95% CI, 0.28–22.3], *p* > 0.71). Recent results from the GARFIELD‐AF registry showed early rhythm control resulted in a lower risk of all‐cause mortality and non‐hemorrhagic stroke [28]. Variables such as age, symptom burden, left atrium size, left ventricular function, and regurgitation influence which management method is more favorable [25]. Furthermore, due to the limited data available comparing different rate control agents for long‐term management, the choice of agent should be based on the underlying substrate and comorbid conditions [25]. In a study of 752 AF patients treated with antiarrhythmic medications, Vinolas et al. reported that obesity was independently associated with a lower probability of pharmacological reversion to sinus rhythm [29]. Yet, despite being an independent risk factor for AF, the guidelines do not consider obesity as a comorbid condition for consideration.

The effect of obesity on certain medications, such as phenytoin, macrolides, glycopeptides, aminoglycosides, fluconazole, and voriconazole, has been well documented [30]. As a result, there is more guidance available on dose adjustments in the obese population, compared to other medications, such as brexpiprazole, despite also being significantly affected by prolonging the time to reach the 90% effective concentration threshold compared to normal weight patients [31].

In contemporary practice, there is growing concern about the use of medications, including antiarrhythmics and anticoagulants in special populations such as those with extreme obesity, with regard to the effectiveness of the treatment [32]. Given the increasing prevalence of concomitant obesity and AF and the toxicity profile of antiarrhythmic drugs, there is a need to understand the pharmacological considerations of obese adults with AF. This is particularly concerning when initial antiarrhythmic clinical trials are older and highly selective, excluding individuals with multimorbidity.

## 2 Aim

This review aimed to describe the evidence of the effect of obesity on the pharmacokinetics of antiarrhythmics in people with AF.

### 2.1 Methods

Three electronic databases including Embase, Medline, and Scopus were searched from inception to June 26, 2023, using key search terms such as “atrial fibrillation,” “obese*,” “overweight,” and “antiarrhythmic” to locate published studies. These databases were selected due to their broad coverage of pharmacological, biomedical, and multidisciplinary literature, encompassing diverse fields and providing a comprehensive range of results. Gray literature was searched using Google Scholar and screening the reference lists of relevant review papers. Refer to Supporting Information S1: Table S1 for a detailed search strategy. Only original research studies that addressed the effect of obesity and body weight on antiarrhythmics in adults with AF were included. Any correspondence, conference abstracts, review papers, letters to the editor/editorials, trial protocols, non‐human studies, and studies published in other than English were excluded. Studies that were not within the target population, intervention, and outcomes were also excluded.

Results were downloaded into EndNote X20.6 [33] citation management software and were deduplicated and screened by title and abstract. Following the initial screening, the remaining records were then uploaded into Covidence® systematic review software [34] for full‐text review and data extraction by two of the authors (F.S. and C.F.). Studies that did not meet design criteria or address the research question were excluded. The results of the search are reported in full and presented in a Preferred Reporting Items for Systematic Reviews and Meta‐Analyses (PRISMA) flow diagram [35], as shown in Supporting Information S1: Figure S1.

A preliminary search of the databases indicated the inappropriateness of conducting a meta‐analysis and risk of bias due to the type and heterogeneity of the studies included. Thus, a narrative review and synthesis approach was adopted. Data were extracted from studies included in the review using a standardized data extraction tool in Covidence. The extracted data included specific details about the study population, study design, intervention (antiarrhythmic drug), and outcomes of significance to the review objective.

## 3 Results

As shown in Supporting Information S1: Figure S1, a total of 6463 articles were initially retrieved. After removing the duplicates (*n* = 1916), 4435 articles were excluded after title and abstract review, leaving 114 articles for full‐text review. Of these, 100 articles were further excluded for reasons summarized in Supporting Information S1: Figure S1, leaving 10 articles that met the inclusion criteria and were included in the narrative synthesis. These 10 articles comprised seven cohort studies (retrospective and prospective), two pharmacokinetic studies, and a single case report.

The sample size of the included articles ranged from 1 to 371 participants who were generally males (41%–85%), aged 59–75 years, and had a BMI of 23–66 kg/m^2^. Over half of the studies were conducted in North America (58%). The remaining studies were conducted in Japan (29%), Austria, France, and Lebanon. The two predominant antiarrhythmics investigated by the studies were amiodarone and dofetilide. Other drugs investigated included diltiazem, flecainide, disopyramide, propafenone, dronedarone, sotalol, vernakalant, and ibutilide. The characteristics of the included studies are summarized in Table 1. Included studies investigated whether the antiarrhythmic drug of interest was affected by obesity and body weight or a superior method of dosing, such as actual body weight versus ideal body weight.

**Table 1:** Study characteristics.

Study	Study design	Sample size	Sex	Mean/median age	Mean BMI/weight	Drug(s)	Results/conclusion
Anderson et al. [36]	Retrospective cohort	*n* = 217	BMI < 30: 81 male (69.8%) BMI ≥ 30: 63 male (62.4%)	Mean: BMI < 30: 69 ± 11.3 BMI ≥ 30: 65.6 ± 8.9	Obese (BMI ≥ 30) Nonobese (BMI < 30)	Dofetilide	Total body weight should be used with caution to calculate initial doses of dofetilide in women compared to ideal body weight.
Cao et al. [37]	Retrospective cohort	*n* = 132	Same dose: 88 (85%) male Different dose: 12 (41%) male	Mean: Same dose: 60 (26–77) Different dose: 70 (46–83)	Same dose: 29.9 (25.1–43.4) kg/m^2^ Different dose: 32.2 (25.3–53.9) kg/m^2^	Dofetilide	ABW‐based dofetilide dosing is reasonable in overweight and obese patients.
Fukuchi et al. [38]	Population pharmacokinetic study	*n* = 23 (151 serum samples)	Male: 18 Female: 5	Mean: 59.2 ± 12.2 (26−82)	BMI (kg/m^2^): 23.8 ± 2.95 (17.6–31.4) Body fat percentage (%): 23.6 ± 4.59 (9.40–37.3)	Amiodarone	The clearance of AMD was influenced by BMI, age and daily dosage of AMD. Population pharmacokinetic analysis confirms that obesity affects the pharmacokinetics of AMD.
Lafuente‐Lafuente et al. [39]	Pharmacokinetic study	*n* = 30	Male: 19 (63.33%)	Mean: 75 ± 14 (35–94)	23.7 ± 4.5 (15–36) kg/m^2^	Amiodarone	No evidence of excessive or unexpected accumulation of amiodarone in adipose tissue on long‐term administration.
Le et al. [40]	Case report	*n *= 1	Male	67	245 kg BMI: 66 kg/m^2^	Amiodarone	Amiodarone's unique pharmacokinetics make high‐dose oral boluses a reasonable strategy for cardioversion of atrial arrhythmias.
Lindmayr et al. [41]	Prospective observational trial	*n* = 316	Male: 195 (61.7%)	Median: 64 (50–73)	26.2 (23.8–29.2) kg/m^2^	Vernakalant, ibutilide	Fixed dose of ibutilide—as compared to the weight‐adapted dose of vernakalant—showed a reduced treatment success with increasing body weight
Ornelas‐Loredo et al. [42]	Observational cohort study	*n* = 311	Obese BMI ≥ 30: 76 (45.2) female Nonobese BMI < 30: 44 (30.8) female	Mean: Obese BMI ≥ 30: 62.4 (12.6) Nonobese BMI < 30: 66.9 (11.2)	Obese, BMI ≥ 30 (*n* = 168): 38.6 (7.5) kg/m^2^ Nonobese, BMI < 30 (*n* = 143): 26.2 (3.6) kg/m^2^	Flecainide, disopyramide, propafenone, amiodarone, dofetilide, dronedarone, sotalol	Obesity may reduce the therapeutic effectiveness of sodium channel blockers
Wang et al. [43]	Retrospective cohort	*n* = 265	Reduced dose: 17 (41%) male Same dose: 143 (64%) male	Mean: Reduced dose: 72 (7) Same dose: 67 (10)	Reduced dose: 30 (6) kg/m^2^ Same dose: 30 (7) kg/m^2^	Dofetilide	Dosing dofetilide using TBW may lead to a greater frequency of dose reduction or discontinuation compared to dosing using ABW or IBW.
Ward et al. [44]	Single‐center retrospective study	*n* = 371	NWB dosing: 116 (54.0%) male WB dosing: 71 (45.5%) male	Median (IQR) NWB dosing: 67 (58–76) WB dosing: 71 (61–79)	Median (IQR) NWB dosing: 30 (26–38) kg/m^2^ WB dosing: 27 (23–30) kg/m^2^	Diltiazem (IV)	There was no significant difference in achieving a therapeutic response between the two strategies. WB dosing approach did result in a greater proportion of patients with an HR < 100 bpm.
Zimmerman et al. [45]	Single‐center retrospective cohort	*n* = 328	Male: 172 (52.4%)	Mean: 69.4 (14.9)	30.3 (8.8) kg/m^2^	Diltiazem (IV)	No difference in the total amount of diltiazem or time to reach goal HR was found in patients according to body weight stratification.

Abbreviations: ABW, actual/adjusted body weight; AMD, amiodarone; BMI, body mass index; HR, heart rate; IBW, ideal body weight; IQR, interquartile range; IV, intravenous; NWB, non‐weight‐based; TBW, total body weight; WB, weight‐based.

### 3.1 Antiarrhythmics Influenced by Obesity and Body Weight

Four studies [38, 39, 40, 42] investigated the effect of obesity and body weight on amiodarone. Fukuchi, Nakashima, and Araki [38] showed that the clearance of amiodarone is influenced by BMI. Interestingly, even though the authors defined obesity based on the body fat percentage (males: > 23%; females: 28%), the average BMI of the included participants was 23.8 kg/m^2^, which would not classify as obese as per the WHO BMI classification. A case report by Le et al. [40] described the use of high oral dose (8000 mg) amiodarone administration in a morbidly obese patient (245 kg) for cardioversion, emphasizing the degree of lipophilicity of amiodarone in obese patients.

In contrast, Lafuente‐Lafuente et al. [39] concluded that there was insufficient evidence to suggest the accumulation of amiodarone in adipose tissue with long‐term use and any amiodarone‐related toxicities because of accumulation in adipose tissue. This was despite their results indicating a higher concentration of amiodarone in the adipose tissue compared with the plasma levels. The study by Ornelas‐Loredo et al. [42], who investigated multiple antiarrhythmics (*n* = 7) in a relatively large population sample (*n* = 311), also opposed earlier results regarding amiodarone. Their initial results indicated that obese (30%) patients performed better than nonobese (6%, difference, 0.24; 95% CI, 0.11–0.37; *p* = 0.001). However, when multivariate analyses were performed, sodium channel blockers and obesity (Class I vs. Class III AAD [obese] odds ratio [OR], 4.54; 95% Wald CI, 1.84–11.20; *p* = 0.001), female sex (OR, 2.31; 95% Wald CI, 1.07–4.99; *p* = 0.03), and hyperthyroidism (OR, 4.95; 95% Wald CI, 1.23–20.00; *p* = 0.02) were significantly associated with failure to respond to antiarrhythmics, which suggests obesity may reduce the therapeutic effectiveness of sodium channel blockers [42]. The authors also found that, unlike obesity, BMI was not associated with failure to respond to Class I antiarrhythmics. However, they proposed that this was likely due to the nonlinearity, large variability, and small sample size at extreme BMI values. Notably, there was disproportionality, with regard to both the number of individual antiarrhythmics (e.g., Class I [flecainide: 85% vs. disopyramide: 2.6%] vs. Class III [amiodarone: 79% vs. dronedarone: 3.6%]) and sample size (Class I: *n* = 115 vs. Class III: *n* = 196).

### 3.2 Antiarrhythmics Not Influenced by Obesity and Body Weight

Four studies [36, 37, 42, 43] investigated the effect of obesity on dofetilide. All four studies were observational, in which three were conducted retrospectively investigating the superior dosing method in obese adults. The findings from these three studies were inconsistent. Cao et al. [37] argued that dofetilide can be dosed based on the current method (i.e. actual body weight), whereas Anderson et al. [36] and Wang et al. [43] concluded that caution should be used when dosing is based on total body weight, especially in females. In contrast, the study by Ornelas‐Loredo et al. [42] reported the influence of obesity only on sodium channel blockers, in agreement with findings from Cao et al. [37].

Two studies [44, 45] investigated diltiazem (IV) dosing methods in different body weights. Ward et al. [44] found no significant difference (66.5% vs. 73.1%; *p* = 0.18) between weight‐based and non‐weight‐based dosing to achieve a therapeutic response. However, patients who were dosed based on weight were more likely to have a heart rate below 100 beats per minute (40.9% vs. 53.5%; *p* = 0.01). Although this suggests that diltiazem is dependent on weight, the authors also found that when stratified based on ideal weight, the weight‐based dosing group achieved a significantly higher incidence of therapeutic response (62.7% vs. 74.3%; *p* = 0.02). Similar results were found by Zimmerman et al. [45] who did not find a significant difference between the total dose (28.7 vs. 34.3 mg; *p* = 0.068) or time (2.3 vs. 2.3 h; *p* = 0.949) to reach the goal heart rate for patients weighing < 100 kg versus ≥ 100 kg.

The remaining antiarrhythmics were investigated in solitary studies. Both sotalol and dronedarone were investigated by Ornelas‐Loredo et al. [42] who claimed these drugs were not affected by obesity. Lindmayr et al. [41] showed that the effectiveness of ibutilide was decreased using fixed doses with increasing body weight (> 60 kg). However, the authors also stated that when adjusted for potential confounders, this effect remained stable (adjusted OR = 0.55 [0.38–0.92]; *p* = 0.02). However, this effect was not seen with vernakalant, where the standard weight‐based dosing method (up to 113 kg) of vernakalant was employed.

## 4 Discussion

Obesity and body weight may potentially affect the pharmacokinetics of only certain antiarrhythmics’, such as amiodarone and sodium channel blockers i.e., flecainide, disopyramide and propafenone.

### 4.1 Amiodarone

The results from two of the four studies in this review showed that the clearance of amiodarone is influenced by BMI, and a higher dose of amiodarone can be given to extremely obese patients (> 40 kg/m^2^). These findings appear to be aligned with other published works [46, 47, 48] that investigated the effect of weight on the pharmacokinetics of amiodarone. It is postulated that the altered pharmacokinetics of amiodarone in obesity may be influenced by reduced hepatic metabolism and increased levels of amiodarone‐binding proteins [32]. Amiodarone has been used for decades to help manage arrhythmia in patients with AF. Together with digoxin, it accounts for 80% of the prescribed medication regimens in developing countries [49]. Despite the history of amiodarone, the pharmacokinetic profile is yet to be truly understood [50]. It is incompletely and erratically absorbed following oral administration. The drug undergoes first‐pass metabolism in the gut wall and/or in the liver and the bioavailability ranges from 22% to 86% [50]. The half‐life of amiodarone ranges from 14 to 110 days but in most cases, it ranges from 14 to 59 days [50]. Due to its markedly lipophilic property and in turn its high Vd (50–100 L/kg), it is extensively distributed in the adipose tissue where it can be up to 125 times that in blood [39, 48, 51]. Consequently, in theory, it can be assumed that morbidly obese patients (> 40 kg/m^2^), who have a larger Vd for amiodarone, would require a higher dose to achieve therapeutic levels [48]. The findings from Lafuente‐Lafuente et al. [39] and Ornelas‐Loredo et al. [42] were the only two studies that contrasted with other studies in this review. Ornelas‐Loredo et al. [42] showed that the therapeutic response to Class III antiarrhythmics (e.g., amiodarone) is similar in both the obese and healthy‐weight patients. Similarly, Lafuente‐Lafuente et al. [39] concluded that there was no evidence to suggest the accumulation of amiodarone in the adipose tissue after long‐term use, but the participants in this study did not qualify as obese according to the WHO criteria (BMI ≥ 30 kg/m^2^).

### 4.2 Sodium Channel Blockers

Sodium channel blockers, including flecainide and propafenone that are lipophilic, have a relatively large Vd (5–13.4 L/kg; and 2.5–4 L/kg, respectively) [52, 53, 54]. However, based on the findings from this review, disopyramide appears to be the opposite of its physiochemical properties, where it has a lower Vd compared to the other two sodium channel blockers (80 L), although details surrounding its lipophilicity are scarce. Despite the wide use of sodium channel blockers, there were only three studies that investigated the effect of obesity and body weight on these drugs. The study by Ornelas‐Loredo et al. [42] was the only one that met the inclusion criteria for this narrative synthesis and showed that obesity and body weight may affect the effectiveness of sodium channel blockers (30% [obese] vs. 6% [nonobese]; *p* = 0.01). There was a reduction in the effectiveness of the Class I antiarrhythmic for every 2.5‐unit increment increase in BMI in patients with obesity. Three additional studies [55, 56, 57] investigated the effect of obesity and body weight on these agents in AF and diabetes and found similar results to that of Ornelas‐Loredo et al. [42]. Sha et al. [32] postulated that obesity affected the inter‐chamber differences in sodium current density and β‐subunit expression, thus causing the reduced efficacy of flecainide in AF [32].

### 4.3 Potassium Channel Blockers

Potassium channel blockers (including dofetilide, sotalol, dronedarone, vernkalant, and ibutilide) were not affected by obesity and body weight. However, when we consider the physiochemical and pharmacokinetic properties, the individual antiarrhythmics have variability in these parameters, where dofetilide is known as a moderate lipophilic drug and has a Vd of ~3.4 L/kg [58, 59]. Dronedarone is known to be less lipophilic compared to amiodarone; however, its Vd is 20 L/kg [59, 60]. These properties would indicate that these two drugs would be affected by obesity and body weight. Wang et al. [61] reached a similar conclusion to Wang et al. [43] where they suggested using ideal body weight instead of actual body weight as may lead to fewer dose reductions or discontinuations, lower peak QTc, and less frequent prolongation of the QTc. On the contrary, a study by Bin Jardan et al. [62] showed that dronedarone metabolic profiles were significantly changed in hyperlipidemia and high‐calorie diets, which may allude to some level of involvement. However, based on the paucity of evidence examining this, it is difficult to draw conclusions regarding the effect.

Unlike the previous two drugs, sotalol is known to be a highly hydrophilic drug and has been shown to have similar pharmacokinetic properties in obese and lean patients [32], despite having low–medium Vd (1.5–2.5 L/kg) [58, 59]. Results from this review indicated that sotalol is unaffected by obesity and body weight. Ornelas‐Loredo et al. [42] showed that sotalol was superior in reducing the percentage of AF burden compared with flecainide. In addition to results from this review, a seminal study by Poirier et al. [63] showed that the pharmacokinetic parameters of sotalol were similar in obese and lean individuals [63]. Although this adds merit to our findings, it is to be noted that like dronedarone, this is based on a singular study; thus, conclusions cannot be drawn.

The two remaining potassium channel blockers (vernakalant and ibutilide) have limited information surrounding both their physiochemical properties and the influence, or lack of influence, of obesity and body weight on these drugs. There was only one additional study [48] that has findings that conflict with those of Lindmayr et al. [41]. Friesen and Ducas [48] showed that although the pharmacokinetic properties of ibutilide were affected by obesity and body weight, this was not to the same degree as amiodarone.

### 4.4 Diltiazem

Unlike the previously discussed antiarrhythmics, diltiazem is used for rate control and is a highly lipophilic drug with a large Vd (3.3 L/kg) [59]. This would suggest diltiazem be affected by obesity and body weight, but this review revealed no difference between weight‐based and actual weight‐based dosing. In contrast, Ward et al. [44] found a weight‐based dosing approach resulted in a greater proportion of patients with an HR < 100 bpm. Our results appear to be consistent with other published studies on this topic, where Ross et al. [64] and Patel et al. [65] also found no difference between a standard diltiazem dosing and weight‐based dosing. Similarly, Erstad et al. [51] found that the same loading dose based on total body weight was effective for both normal and morbidly obese patients.

### 4.5 Influence of Lipophilicity on Pharmacokinetics

It has been hypothesized that the lipophilicity of drugs may influence their pharmacokinetics in patients with obesity [22]. However, it is challenging to predict which antiarrhythmics may be influenced by obesity and body weight based solely on the physiochemical and pharmacokinetic properties of the drug. The discrepancy in the impact of obesity and body weight on the physiochemical and pharmacokinetic properties can be seen in the case of amiodarone versus other lipophilic antiarrhythmics (i.e., diltiazem, dofetilide, and dronedarone). Interestingly, hydrophilic antiarrhythmics, such as sotalol and digoxin [51, 66, 67, 68], appear to consistently not be influenced by obesity and body weight.

These findings suggest that obesity and body weight may influence the pharmacokinetics of extremely lipophilic drugs, such as amiodarone and Class I antiarrhythmics, but this influence is not limited just to the level of lipophilicity. Other factors, such as protein binding and regional blood flow, may also play a role [69]. This assumption is based on heterogeneous observational studies reporting BMI and may also be subject to confounding factors such as concomitant medications/conditions, obesity class, and route of administration. The controversy surrounding the validity of BMI as a mode of assessment should also be noted. In the setting of a lack of guidance from the clinical practice guidelines and published clinical outcomes within the individuals with obesity, clinicians prescribe based on the standard recommended dosing regimens [51]. This is why contemporary reviews, such as by Sha et al. [32], challenge the current European Society of Cardiology guidelines [70], which recommend using Class I antiarrhythmics over Class III in the obese patient subgroup [32]. Larger scale observational studies or registries, which include all the antiarrhythmics may provide more strength to the existing literature on this topic, but findings will still be subject to confounding factors.

## 5 Limitations

This study has several limitations. The included studies were restricted to English language and human studies focusing on AF as the condition of interest, which may have limited the number of eligible studies. This restriction aimed to minimize the implications of the results on AF and to enable an examination of obesity as the variable of interest. Additionally, the majority of the included studies were observational in design, making them subject to confounding factors as discussed. Given the lack of published randomized controlled trials on this topic and the inability to combine the data for a meta‐analysis, the results from this review are primarily intended to hypothesis generation for future studies. As we enter an era of both multimorbidity and polypharmacy focusing on drug effects in isolation and combination is critically important.

In light of the increasing societal burden of both AF and obesity and the higher likelihood of an individual concomitantly having both of these conditions, a pragmatic approach incorporating large‐scale head‐to‐head randomized trials examining both the clinical and pharmacokinetic outcomes of antiarrhythmics is warranted [71, 72]. Although underweight, cachexia, sarcopenia, and frailty were not within the scope of this review, closer examination of the evidence related to dosing of antiarrhythmics is needed in the context of comorbidities. As there is an increasing focus on frailty, considering underweight is likely to be an important focus in the future.

## 6 Implications

The presence of multimorbidity in patients with AF, such as diabetes and obesity, underscores the challenge of polypharmacy in clinical practice. Coupled with the complexity of obese patients, this necessitates a move toward personalized care strategies that consider individual patient characteristics, including pharmacokinetic variations influenced by comorbidities. Although risk prediction models offer some insight, their limited guidance underscores the need for more nuanced approaches to treatment decisions. In this context, the potential role of automated clinical decision support systems [73, 74] emerges as a promising avenue to assist clinicians in navigating the complexities of polypharmacy and multimorbidity, thereby optimizing patient outcomes.

The findings from this review provide the foundations for further research, which in turn can have implications for future clinical practice guidelines and hospital policies and protocols to manage high‐risk medications, such as antiarrhythmics, in special and complex population groups including frail and obese individuals. Furthermore, because obesity is a risk factor for multiple morbidities—such as cardiovascular disease, diabetes, sleep apnea, and cancer [75]—this may also have implications on the current methods of developing clinical guidelines and the healthcare system to manage patients with multiple complex comorbidities [76].

## 7 Conclusion

Not all antiarrhythmics appear to be affected equally by obesity and body weight. Instead, factors such as a drug's level of lipophilicity may also influence the pharmacokinetics of the drug and the need for dose modification. Further research is needed to confirm the clinical significance of these findings and to develop guidance methods that can be used by clinicians and other healthcare professionals to select antiarrhythmics and calculate doses that are individualized and appropriate for the obese–AF population.

## Author Contributions

F.S. and C.F. conceptualized the study. F.S., R.W., and C.F. developed the search strategy. F.S. and C.F. screened and reviewed articles. F.S., R.W., R.L.C., P.M.D., S.C.I., and C.F. wrote and edited the manuscript. All authors reviewed and approved the final version.

## Conflicts of Interest

C.F. was a coauthor of the National Heart Foundation of Australia & Cardiac Society of Australia & New Zealand's Australian Clinical Guidelines for the Diagnosis and Management of Atrial Fibrillation (2018). All other authors declare no conflicts of interest.

## Supporting information

Supporting information.

## Data Availability

Data sharing is not applicable to this article as no new data were created or analyzed in this study.
