# Supplementary material for: Effect of Obesity on the Use of Antiarrhythmics in Adults With Atrial Fibrillation: A Narrative Review
Source: Clin Cardiol. 2024 Aug 21;47(8):e24336. doi: 10.1002/clc.24336 (PMC11339320; doi:10.1002/clc.24336)
Supplement: Supplementary file 1 — Supporting information. [file CLC-47-e24336-s001.docx]

Supplementary Material

Table 1: Search Strategy

| Database | Search Terms | Results |
| --- | --- | --- |
| Scopus | ( TITLE-ABS-KEY ( "atrial fibrillation" ) OR TITLE-ABS-KEY ( "atrial flutter" ) OR TITLE-ABS-KEY ( "auricular fibrillation" ) OR TITLE-ABS-KEY ( "atrium fibrillation" ) OR TITLE-ABS-KEY ( af ) OR TITLE-ABS-KEY ( "a* fibrillation* " ) ) AND ( TITLE-ABS-KEY ( "Morbid Obesity" ) OR TITLE-ABS-KEY ( "Binge-Eating Disorder" ) OR TITLE-ABS-KEY ( obesity ) OR TITLE-ABS-KEY ( "obes*" ) OR TITLE-ABS-KEY ( overweight ) OR TITLE-ABS-KEY ( "body weight" ) OR TITLE-ABS-KEY ( "weight gain" ) OR TITLE-ABS-KEY ( "body mass index" ) OR TITLE-ABS-KEY ( "abdominal fat" ) OR TITLE-ABS-KEY ( "Obesity Paradox" ) OR TITLE-ABS-KEY ( "Maternal Obesity" ) OR TITLE-ABS-KEY ( "Pediatric Obesity" ) OR TITLE-ABS-KEY ( "Obesity Management" ) OR TITLE-ABS-KEY ( "Abdominal Obesity" ) OR TITLE-ABS-KEY ( "Hypoventilation Syndrome" ) OR TITLE-ABS-KEY ( "Metabolically Benign" ) OR TITLE-ABS-KEY ( obesity ) OR TITLE-ABS-KEY ( "Anti-Obesity Agents" ) OR TITLE-ABS-KEY ( "over weight" ) OR TITLE-ABS-KEY ( "fat overload syndrom*" ) OR TITLE-ABS-KEY ( "weight adj*" ) OR TITLE-ABS-KEY ( "weight chang*" ) OR TITLE-ABS-KEY ( "waist-hip ratio" ) OR TITLE-ABS-KEY ( bmi ) OR TITLE-ABS-KEY ( "overweigh*" ) OR TITLE-ABS-KEY ( corpulent ) OR TITLE-ABS-KEY ( "adipos*" ) OR TITLE-ABS-KEY ( "Brown Adipose Tissue" ) OR TITLE-ABS-KEY ( adipocytes ) OR TITLE-ABS-KEY ( "Adipose Tissue" ) OR TITLE-ABS-KEY ( adiposity ) OR TITLE-ABS-KEY ( adipocyte ) ) AND ( TITLE-ABS-KEY ( antiarrhythmic ) OR TITLE-ABS-KEY ( "Anti-Arrhythmia Agents" ) OR TITLE-ABS-KEY ( "dysrhythmic agent" ) OR TITLE-ABS-KEY ( dronedarone ) OR TITLE-ABS-KEY ( amiodarone ) OR TITLE-ABS-KEY ( disopyramide ) OR TITLE-ABS-KEY ( dofetilide ) OR TITLE-ABS-KEY ( azimilide ) OR TITLE-ABS-KEY ( ibutilide ) OR TITLE-ABS-KEY ( flecainide ) OR TITLE-ABS-KEY ( propafenone ) OR TITLE-ABS-KEY ( quinidine ) OR TITLE-ABS-KEY ( cibenzoline ) OR TITLE-ABS-KEY ( moricizine ) OR TITLE-ABS-KEY ( mexiletine ) OR TITLE-ABS-KEY ( procainamide ) OR TITLE-ABS-KEY ( sotalol ) OR TITLE-ABS-KEY ( esmolol ) OR TITLE-ABS-KEY ( digoxin ) OR TITLE-ABS-KEY ( verapamil ) OR TITLE-ABS-KEY ( diltiazem ) OR TITLE-ABS-KEY ( "Non-dihydropyridine* " ) OR TITLE-ABS-KEY ( "calcium channel blocker* " ) OR TITLE-ABS-KEY ( "Adrenergic beta-Antagonists" ) OR TITLE-ABS-KEY ( "beta blocker" ) OR TITLE-ABS-KEY ( "Beta adrenoceptor antagonists" ) OR TITLE-ABS-KEY ( "Membrane-active rhythm-control" ) OR TITLE-ABS-KEY ( metoprolol ) OR TITLE-ABS-KEY ( ajmaline ) OR TITLE-ABS-KEY ( sparteine ) OR TITLE-ABS-KEY ( lidocaine ) OR TITLE-ABS-KEY ( tocainide ) OR TITLE-ABS-KEY ( "sodium channel blocker" ) OR TITLE-ABS-KEY ( carvedilol ) OR TITLE-ABS-KEY ( propranolol ) OR TITLE-ABS-KEY ( atenolol ) OR TITLE-ABS-KEY ( bisoprolol ) OR TITLE-ABS-KEY ( nebivolol ) OR TITLE-ABS-KEY ( "potassium channel blocker" ) OR TITLE-ABS-KEY ( vernakalant ) ) | 1866 |
| MEDLINE (OVID) | 1. "atrial fibrillation".ab,ti.  2. exp Atrial Fibrillation/  3. exp Atrial Flutter/  4. "atrial flutter".ab,ti.  5. "auricular fibrillation".ab,ti.  6. "atrium fibrillation".ab,ti.  7. AF.ab,ti.  8. "a* fibrillation* ".ab,ti.  9. 1 or 2 or 3 or 4 or 5 or 6 or 7 or 8  10. exp Obesity, Morbid/ or exp Binge-Eating Disorder/ or exp Obesity/  11. "obes*".ab,ti.  12. overweight.ab,ti.  13. exp Overweight/  14. exp Body Weight/  15. "body weight".ab,ti.  16. "weight gain".ab,ti.  17. exp Weight Gain/  18. exp Body Mass Index/  19. "body mass index".ab,ti.  20. "abdominal fat".ab,ti.  21. exp Abdominal Fat/  22. exp Obesity Paradox/ or exp Obesity, Maternal/ or exp Pediatric Obesity/ or exp Obesity Management/ or exp Obesity, Abdominal/ or exp Obesity Hypoventilation Syndrome/ or exp Obesity, Metabolically Benign/ or exp Obesity/ or exp Anti-Obesity Agents/ or exp Obesity, Morbid/  23. "over weight".ab,ti.  24. "fat overload syndrom* ".ab,ti.  25. "weight adj*".ab,ti.  26. "weight chang* ".ab,ti.  27. "waist-hip ratio".ab,ti.  28. exp Waist-Hip Ratio/  29. BMI.ab,ti.  30. "overweigh* ".ab,ti.  31. corpulent.ab,ti.  32. "adipos*".ab,ti.  33. exp Adipose Tissue, Brown/ or exp Adipocytes/ or exp Adipose Tissue/ or exp Adiposity/  34. adipocyte.ab,ti.  35. 10 or 11 or 12 or 13 or 14 or 15 or 16 or 17 or 18 or 19 or 20 or 21 or 22 or 23 or 24 or 25 or 26 or 27 or 28 or 29 or 30 or 31 or 32 or 33 or 34  36. antiarrhythmic.ab,ti.  37. exp Anti-Arrhythmia Agents/  38. "dysrhythmic agent".ab,ti.  39. dronedarone.ab,ti.  40. exp Dronedarone/  41. exp Amiodarone/  42. amiodarone.ab,ti.  43. disopyramide.ab,ti.  44. exp Disopyramide/  45. dofetilide.ab,ti.  46. azimilide.ab,ti.  47. ibutilide.ab,ti.  48. flecainide.ab,ti.  49. exp Flecainide/  50. exp Propafenone/  51. propafenone.ab,ti.  52. quinidine.ab,ti.  53. exp Quinidine/  54. cibenzoline.ab,ti.  55. moricizine.ab,ti.  56. exp Moricizine/  57. exp Mexiletine/  58. mexiletine.ab,ti.  59. procainamide.ab,ti.  60. exp Procainamide/  61. exp Sotalol/  62. sotalol.ab,ti.  63. esmolol.ab,ti.  64. digoxin.ab,ti.  65. exp Digoxin/  66. exp Verapamil/  67. verapamil.ab,ti.  68. diltiazem.ab,ti.  69. exp Diltiazem/  70. "Non-dihydropyridine* ".ab,ti.  71. "calcium channel blocker* ".ab,ti.  72. exp Calcium Channel Blockers/  73. exp Adrenergic beta-Antagonists/  74. "beta blocker".ab,ti.  75. "Beta adrenoceptor antagonists".ab,ti.  76. "Membrane-active rhythm-control".ab,ti.  77. metoprolol.ab,ti.  78. exp Metoprolol/  79. exp Ajmaline/  80. Ajmaline.ab,ti.  81. Sparteine.ab,ti.  82. exp Sparteine/  83. exp Lidocaine/  84. Lidocaine.ab,ti.  85. Tocainide.ab,ti.  86. exp Tocainide/  87. exp Sodium Channel Blockers/  88. "sodium channel blocker".ab,ti.  89. Carvedilol.ab,ti.  90. exp Carvedilol/  91. exp Propranolol/  92. Propranolol.ab,ti.  93. Atenolol.ab,ti.  94. exp Atenolol/  95. exp Bisoprolol/  96. Bisoprolol.ab,ti.  97. Nebivolol.ab,ti.  98. exp Nebivolol/  99. exp Potassium Channel Blockers/  100. "potassium channel blocker".ab,ti.  101. Vernakalant.ab,ti.  102. 36 or 37 or 38 or 39 or 40 or 41 or 42 or 43 or 44 or 45 or 46 or 47 or 48 or 49 or 50 or 51 or 52 or 53 or 54 or 55 or 56 or 57 or 58 or 59 or 60 or 61 or 62 or 63 or 64 or 65 or 66 or 67 or 68 or 69 or 70 or 71 or 72 or 73 or 74 or 75 or 76 or 77 or 78 or 79 or 80 or 81 or 82 or 83 or 84 or 85 or 86 or 87 or 88 or 89 or 90 or 91 or 92 or 93 or 94 or 95 or 96 or 97 or 98 or 99 or 100 or 101  103. 9 and 35 and 102 | 421 |
| Embase (OVID) |  | 4012 |

Table 2: Pharmacokinetic properties of studied antiarrhythmics; TPC: Time to peak concentration, BA: Bioavailability; IR: immediate release, CR: controlled release

| **Drug** | **Absorption** | **Lipophilicity (Log P)^24^** | **Distribution (Vd)** | **Primary excretion site** | **Elimination Half Life** |
| --- | --- | --- | --- | --- | --- |
| Amiodarone^1-3^ | TPC: 3-7 hrs (oral)  BA: ~50% | Extremely High  (7.24-7.64) | 60 L/kg | Bile | chronic dosing:  26 to 107 days |
| Dofetilide^4^ | TPC: 1-3 hrs (oral)  BA: >90% | Low (0.24-2.17) | 3-4 L/kg | Kidney (~80%) | 7.5-10 hrs |
| Vernakalant^5,6^ | BA: 58-71% (oral) | Moderate (2.74-2.51) | 2 L/kg | Kidney | 3-5.5 hrs |
| Ibutilide^7^ | N/A | Moderate-High  (2.54-4.72) | 9-13 L/kg | Kidney (80-82%) | 2-12 hrs |
| Flecainide^8,9^ | TPC: 1-6 hrs (oral) | Moderate-High  (2.98-3.78) | 4.9-10 L/kg | Kidney: 10% to 50% | 12-27 hrs |
| Disopyramide^10-12^ | TPC: 2 hrs (IR), 4.9 +/- 1.4 hrs (CR)  BA: 80% (oral) | Moderate-High  (2.58-3.47) | 90 to 100 L | Kidney (50% unchanged; 20% as metabolite) | 4-10 hrs |
| Propafenone^13^ | TPC: 3-8 hrs (oral)  BA: 3.4-10.6% (IR) | Moderate-High  (3.1-3.54) | ~ 252 L | Kidney (~50% as metabolite) | 2-10 hrs |
| Dronedarone^14,15^ | TPC: 3-6 hrs (oral)  BA: 4% (oral) | Very High (4.63- 6.46) | 1400 L | Faecal (84%) | 13-19 hrs |
| Diltiazem^16-20^ | TPC: 2-4 hrs (IR), 10-18 hrs (CR)  BA: 40% (IR), >90% (CR) | Moderate (2.73-2.8) | 305-391 L | Kidney & Bile | IR: 3-4.5 hrs  CR: 4-9.5 hrs |
| Sotalol^21-23^ | TPC: 2.5-4 hrs (oral)  BA: 90-100% (oral) | Moderate Hydrophilic to Low Lipophilic (-0.4-0.85) | 1.6-2.4 L/kg | Kidney: 66-88% unchanged | 12 hrs |

**Identification of Studies**

Duplicate records removed (n = 1,916)

Records identified from:

**Databases (n = 6,299)**

- Scopus (n = 1,866)
- MEDLINE (OVID) (n = 421)
- Embase (OVID) (n = 4,012)

**Grey Literature (n = 164)**

**Identification**

Records excluded (n = 4,435)

Records screened

(n = 4,547)

Reports not retrieved (n = 0)

Reports sought for retrieval

(n = 114)

**Screening**

Reports excluded **(n = 104)**:

Wrong patient population (n = 41)

Wrong study design (n = 33)

Wrong intervention (n = 14)

Not in English Language (n = 6)

Wrong outcomes (n = 9)

Duplicate dataset (n = 1)

Reports assessed for eligibility

(n = 114)

Studies included in review

(n = 10)

**Included**

Figure 1: PRISMA Diagram

**Supplemental References:**

1. Wyeth Pharmaceuticals Inc. Product Information: CORDARONE(R) oral tablets, amiodarone oral tablets. In: Philadephia; 2018.

2. Wyeth Laboratories. Product Information: Cordarone(R) amiodarone HCl tablets. In: Philadelphia; 2004.

3. Wyeth Pharmaceuticals Inc. Product Information: Cordarone(R) oral tablets, amiodarone HCl oral tablets. In: Philadelphia; 2009.

4. Pfizer Labs. Product Information: TIKOSYN(R) oral capsules, dofetilide oral capsules. In: New York; 2019.

5. D F. Vernakalant (RSD1235): a novel, atrial-selective antifibrillatory agent. *Expert Opin Investig Drugs*. 2007;16:519-532.

6. Cardiome UK Limited. Product Information: BRINAVESS intravenous injection concentrated solution, vernakalant HCl intravenous injection concentrated solution. In: Middlesex, United Kingdom; 2015.

7. Pharmacia & Upjohn Company. Product Information: Corvert(R), ibutilide fumarate injection. In: Kalamazoo, Michigan; 2002.

8. Roxane Laboratories Inc. Product Information: flecainide acetate oral tablet, flecainide acetate oral tablet. In: Columbus, Ohio; 2003.

9. Tjandra-Maga TB VR, Van Hecken A, Mullie A, De Schepper PJ. Flecainide: single and multiple oral dose kinetics, absolute bioavailability and effect of food and antacid in man. *Br J Clin Pharmacol*. 1986;22:309-316.

10. G.D. Searle & Company. Product Information: NORPACE(R) Oral Capsule and NORPACE CR(R) Oral Extended-Release Capsule, disopyramide phosphate oral capsule, extended-release capsule. In: Chicago; 2001.

11. Karim A. The pharmacokinetics of Norpace(R). *Angiology*. 1975;26:85-98.

12. Searle Pharmaceuticals. Product Information: Norpace(R), disopyramide. In: Chicago; 1995.

13. Reliant Pharmaceuticals. Product Information: Rythmol(R) SR, propafenone. In: Liberty Corner, NJ; 2003.

14. Sanofi-aventis US LLC. Product Information: MULTAQ(R) oral tablets, dronedarone oral tablets. In: Bridgewater, NJ; 2017.

15. Sanofi-aventis U.S. LLC. Product Information: MULTAQ(R) oral tablets, dronedarone oral tablets. In: Bridgewater, NJ; 2009.

16. Valeant Pharmaceuticals North America LLC. Product Information: CARDIZEM(R) oral tablets, diltiazem HCl oral tablets. In: Bridgewater, NJ; 2014.

17. Bausch Health US LLC. Product Information: CARDIZEM(R) CD oral extended-release capsules, diltiazem HCl oral extended-release capsules. In: Bridgewater, NJ; 2020.

18. Abbott Laboratories. Product Information: CARDIZEM(R) LA extended-release oral tablets, diltiazem hydrochloride extended-release oral tablets. In: North Chicago; 2010.

19. Bedford Laboratories. Product Information: diltiazem hydrochloride intravenous solution, diltiazem hydrochloride intravenous solution. In: Bedford, Ohio; 2005.

20. Andrx Pharmaceuticals Inc. Product Information: CARTIA XT(TM) oral extended-release capsule, diltiazem hydrochloride oral extended-release capsule. In: Lauderdale, FL; 2003.

21. Arbor Pharmaceuticals. Product Information: SOTYLIZE(TM) oral solution, sotalol HCl oral solution. In: Atlanta; 2014.

22. Uematsu T KM, & Nakashima M. Comparative pharmacokinetic and pharmacodynamic properties of oral and intravenous (+)-sotalol in healthy volunteers. *J Pharm Pharmacol*. 1994;46:600-605.

23. Arrow Pharma Pty Ltd. Australian Product Information – Sotacor (Sotalol Hydrochloride) Tablets. In: Cremorne, VIC; 2023.

24. Knox C, Wilson M, Klinger CM, et al. DrugBank 6.0: the DrugBank Knowledgebase for 2024. Nucleic Acids Res. 2024 Jan 5;52(D1):D1265-D1275. doi: 10.1093/nar/gkad976.
